# An examination of the design for a prefabricated housing unit in Cyprus in terms of energy, daylighting and cost

**DOI:** 10.1038/s41598-023-38045-5

**Published:** 2023-08-03

**Authors:** Andreas Savvides, Aimilios Michael, Constantinos Vassiliades, Despina Parpa, Elina Triantafyllidou, Maria Englezou

**Affiliations:** 1 Department of Architecture, University of Cyprus, Nicosia, Cyprus; 2 Department of Architecture, Land and Environmental Sciences, Neapolis University Pafos, Pafos, Cyprus

**Keywords:** Environmental sciences, Environmental impact, Solar energy, Engineering

## Abstract

Housing prefabrication emerged as an energy and cost-saving solution, which can also be linked to the reduction of environmental impacts, as well as to the development of green construction practices. In the first part of this study, a comprehensive literature review of the prefabricated assembly methods and their inherent potential, in terms of both design and construction are presented. Design strategies that incorporate the integration of environmental systems are also considered. A classification and taxonomy of archetypes is included, based on key design principles pertaining to environmental design. Based on the conclusions drawn from the assessment of these considerations, this paper revisits the realm of design and construction techniques used in energy efficient and environmentally compatible prefabricated housing unit in a Cypriot context. Cost-saving strategies are proposed, as well as architectural design and fabrication recommendations. Consequently, the research aims to contribute to existing literature by drawing results from an actual demonstration project in Cyprus. It goes on to outline considerations affecting the front end of the design and construction processes based on criteria for efficient prefabrication and modular construction. Findings are then related to simulations for energy and daylighting performance supplemented by a techno-economic analysis, aiming to demonstrate the viability of this enterprise. In this way it is hoped that stakeholders considering the adoption of this approach to design and construction may make better informed and more appropriate decisions.

## Introduction

Prefabrication in housing entails the fabrication of building components in a production facility rather than on an actual site, and then transportation to a given lot for assembly^1–3^. In the case of traditional construction some of the building components are manufactured goods, such as certain partitions, ceiling or flooring parts, while what are termed prefabricated houses tend to involve larger building components, such wall panels or entire housing components walled in room volumes. Other modules that are constructed whole may have a specific use, such as bathrooms or kitchens. Yet, in other instances entire houses are manufactured in factory assembly yards before being transported to the building site and hooked up to services to become instantly functioning^4^.

Architectural historians in the United States date housing prefabrication and transportation of components in that context to nearly four hundred years ago and to the shipment of wood paneled housing components to Cape Ann in Massachusetts in the 1620s, to provide shelter for fishermen in the area^5^. In the same timeframe, similar techniques for the quick erection of log cabins were introduced across the Atlantic Ocean in Sweden. Other similar examples, saw the beginning of the production of housing kits-of-parts that could be packed and shipped in standardized freight aboard carriages, ships, trains, automobiles and eventually airplanes^5^.

This experimentation into systematized and transportable housing, culminated in the early twentieth century with the Sears Roebuck prefabricated homes that could be picked out of catalogues purchased by the company’s subscribers starting in 1908^6^. This practice proved of interest to a number of well-known architectural personalities such as, Le Corbusier, Walter Gropius, Frank Lloyd Wright, Jean Prouvé and Paul Rudolph, amongst others, who unpacked the issue of housing shortages. This was especially true after the second world war, where innovative use of this evolving housing production technique saw extensive use, despite criticism received in subsequent years by users who sought individuality and freedom of choice^7^.

Housing production at an industrial scale has been of concern to builders and fabricators since the mid-nineteenth century; and the beginnings of the industrial revolution. With the advent of the assembly line, a number of architects and engineers tried to tackle the housing shortage problem by introducing mass production of housing components^7,8^. An example of such an attempt was Buckminster Fuller’s Dymaxion House^9^. The technology used was based on fitting various groups of distinct building components into sub-assemblies that constitute parts of larger modular structures, such as door, window or wall assemblies. Assembly line techniques, as used in other industries, may then be produced and put together to form entire prefabricated units, which may be transported to the site and assembled there on more permanent foundations^7^.

The evolution of the typology in the late twentieth and early twenty-first centuries has seen customization and spatial flexibility as significant requirements related to the assembly line techniques. These in turn, have been augmented by the introduction and wider use of computer-aided design and manufacturing processes, which furtherenable designers, builders and even users to configure an increased number of options and permutations of the basic modular structure. Although this further facilitates economies of scale, by engaging in the production of larger quantities of repetitive components more cheaply, it also addresses aspects of cultural production and adds to the narrative of mass production, by introducing customers as a part of the stakeholders’ team in the assembly line and as co-generators in the design process^10^.

Research has also shown that prefabricated systems can reduce construction time and labour costs^11^ that will eventually result in cost savings^12^. However, it has also been suggested that their initial cost can be higher than traditional construction methods due to the need for specialized manufacturing and transportation of the building components^11,13,14^.

Literature also addresses the energy, daylighting and cost considerations that may help identify additional benefits, but also limitations of these systems^15–17^. It is shown, that prefabricated housing systems can improve energy efficiency by reducing air infiltration and heat loss, as well as by incorporating insulation and air-tight building envelopes^18^. In addition, prefabricated systems can be designed with passive solar features to improve not only energy performance, but also the provision of daylight^19^. Optimization of natural light penetration, may improve indoor visual comfort and reduce the need for artificial lighting^20^. This is supported by the prefabricated systems’ ability for transformation, which facilitates the incorporation of architectural components such as skylights, clerestory windows and light shelves^21^.

Consequently, we may safely assume that careful consideration of the specific design attributes inherent in prefabricated buildings may ensure building systems effectiveness and cost-efficiency. Thus, the current research takes a first step in identifying best practices and optimal design strategies for the evolution of prefabricated housing systems that might serve the Cypriot and perhaps by extension in the Eastern Mediterranean housing development context.

### Principles of prefabrication

Prefabrication, in the context of housing production, tends to entail the development of building modules or systems that are the result of industrialized procedure^22^. Reference is also usually made to the employment of more recent and contemporary methods of fabrication (at a factory line), transportation (to site) and assembly (onsite)^23^. In this investigative framework, this paper will propose a taxonomy of this building typology, by examining primarily panelised prefabrication in conjunction with a kit-of-parts making use of metal components. Further, a comparison will be effected between prefabrication and conventional building construction making use of alternative materials for cladding^24^.

The principles of prefabrication are also seen in a broader design context wherein efficiencies are sought in improving the environmental performance and building life cycle of prefabricated structures from assembly to disassembly or even to demolition^25,26^. Customization and flexibility of use have also been seen as advantageous, with panelization lauded for its ability to address aspects of insulation performance as a result of ever more precise fabrication techniques. When coupled to smart user-comfort monitoring, these systems may improve user occupancy quality, while lowering energy demand and reducing the energy costs for the household^27–29^. Other factors that play out in the story of prefabricated housing, but which will not be dealt with directly in this paper, may include aspects of: i. waste management (both during the fabrication process of the various housing components and at the end of the prefabricated structure’s useful life cycle)^30,31^, ii. reduced construction time onsite through ease of assembly, and last but not least, iii. the potential for minimizing disruption in the everyday life of a community associated with the dirt and noise of a building construction site^27^.

### Regional challenges in housing unit prefabrication

This paper looks into the challenge faced by these localities that may be more remote and further away from the traditional prefabrication research and industrial production centres in Sweden, Germany, the Netherlands or even Japan further afield that sport highly developed and craft-based industries.

#### A humanitarian crisis

The premise in Cyprus, itself located in the Levantine Basin of the eastern Mediterranean, has been a demonstration project to tackle just such challenges. The initiative has come about as a proposal to house this recent influx of transient-turned-more-permanent populations, fleeing war, poverty and natural disasters—primarily in this case from the Middle East and further afield, and also from Africa. Most of these people would like to make their way to what they perceive as the more prosperous and politically stable European continent via gateway nations such as Cyprus. However, border closures in continental Europe have forced many of them to remain in what are often termed “welcome centres” and unable to move on to their desired destinations.

As a consequence, local knowhow in the prefabrication of mass housing needs to evolve fast, by adapting best practices from more advanced industrialized nations and by considering the local state of the art if such parameters as efficiency and ease and speed of assembly are to be met, so as to move these people out of tents and in to more permanent housing quarters^32,33^.

In doing so, this investigation highlights circumstances that may vary from those in continental European nations and related more to their island chains or to smaller island countries in the region. The information and data collected and utilized, is focused more on local dynamics and on the examination of empirical data from a small island country^34,35^. As such, the approach to the challenge of housing people at a time of crisis in an appropriate, decent, safe, timely and inexpensive fashion may be relevant and it may indeed find resonance with other locales of a similar scale, similar climatic conditions, and similar levels of industrial prefabrication capacity, certainly across the Mediterranean, but also elsewhere similar expectations need to be met.

#### The state of the building sector in the SEMENA Region and the strive for sustainable development

In addition to the question of the evolution of housing prefabrication, the building sector in the SEMENA (region of Southeastern Europe, the Middle East and North Africa), also has to contend with an ever-growing energy demand certainly in the 2020s^36^. It is worth mentioning that, both the World Bank and the International Energy Agency (IEA) have proceeded to estimate that the anticipated energy demands of developing countries are expected to double over the next 40 years^37^. This is the result of intense urbanization and local population growth in a range that spans 2–6%, according to the UN Habitat, augmented by the influx of economic and political migrants, asylum seekers and refugees (UN^38^).

The discussion on the production of sustainable housing is not recent, and it has been ongoing for a few years now, especially in the form of net-zero energy buildings or NZEBs, as noted and debated by a number of researchers^39–42^. However, regional investigations considering contemporary emergencies that have created a new urgency that looks to make the most of combining the advantages of prefabrication with building integrated energy systems—in this case, solar—are not extensive or definitive^28,29,43,44^

It has also been noted that any such projects going through a process of energy and environmental design evaluation, such as LEED, results in a cost premium of some 10% with regards to construction costs^45^, while an opposing view notes that energy savings in the range of 30–55% are effected by prefabricated units, as compared to conventional buildings^46^. This, it is argued, is possible, when one considers operational expenses over time (in the building’s life cycle) and as a result of more energy-efficient structures. Also, other non-cost related benefits assigned to “green” buildings need to be considered when evaluating such typologies^47^. One such benefit has to do with the inhabitants’ comfort, which can result from the optimization of the regulation of the dynamic equation that controls values of temperature and humidity^48–50^ and which has major attributes relating to high indoor environmental quality (IEQ)^47,51^.

#### The prefabrication conundrum for housing authorities and housing development agencies

Housing authorities, which have adopted and show a preference for the utilization of prefabrication techniques, have reported that preassembly at the production line—given extensive use of the same jigs and moulds, essentially unmodified and without undue modifications—lead not only to higher efficiency in construction but also enhanced quality control and better workmanship^52^. Typical components in this framework have so far included building components such as, flooring and ceiling decks, roof parapets, partition walls and façade panels, as well as vertical circulation components such as staircases and specialized modules such as kitchens and bathrooms with accompanying water tanks.

Further reports, from similar sources, include dealing with issues of quality control at the site and also related aspects of buildability and to pre-and-post occupancy efficiencies, in terms energy utilization and waste management. Another challenge is ensuring a more streamlined and cleaner environment for most of the building trades involved on site, as a result of choosing this method of construction^53^. In some localities such as in Singapore, a first has been achieved by the introduction of guidelines and scoring systems that quantify buildability under the Buildable Design Appraisal System (BDAS) when assessing developments of this typology^53,54^. Yet other empirical studies that support this observation indicate a significant correlation between buildability and the aforementioned efficiencies in housing production and quality control, especially as clients assume key roles in prefabrication technology adoption and implementation that in turn drive innovation in the building sector^1,2^.

### Prefabrication use: the case of Cyprus

In Cyprus, the prefabricated building industry is still at an early stage of development, leading to prefabricated housing structures usually being constructed with low-cost materials and conventional construction methods. The units themselves are primarily used as secondary or ancillary structures^27,55^. During the severe economic downturn of the past decade that also affected Cyprus, the interest in prefabricated houses increased, mainly due to the low cost of this type of structure compared to what is offered by conventional construction.

This trend persists even after a rebound in the economy, but unfortunately, without any significant improvements in output^27^. A survey was conducted by Michael et al.^55^, which was based on data obtained by questionnaires filled by companies exclusively engaged in building prefabrication. The questions relate to the average number of sales, the preferred type/use of construction, the cost and the required delivery time, as well as the preferred size for the units for the years 2011–2014. It is noted that the dominant use was for offices and ancillary buildings, while housing took third place.

In the housing sector, the evaluation of the questionnaires revealed that in 2011 each company proceeded with the construction of an average of 17 permanent or occasional housing units, an average of 12 in 2012, an average of 9 in 2013 and an average of 10 in 2014. The differentiation noted in sales figures for the period from 2012 to 2014, as compared to 2011, reflects the overall period of the economic crisis in Cyprus^55^.

The average cost and delivery time for the 165 prefabricated units is noted as 560 euros per m^2^ and 5.5 weeks respectively for permanent houses, 490 euros per m^2^ and 5 weeks for vacation homes, 350 euros per m^2^ and 3.5 weeks for offices and 300 euros per m^2^ and 3 weeks for warehouses and ancillary buildings. The preferred size of prefabricated housing units by Cypriot buyers is about 50–75 m^2^ per housing unit, followed by a preference for housing units in the range of 75–100 m^2^. Following this survey, further research work was focused on the design, fabrication and assembly parameters for an autonomous modular and spatially flexible housing unit. The unit adheres to principles of prefabrication and offers an improved alternative to what local industry currently has on offer in Cyprus that shows similarities to challenges faced by other Mediterranean islands or island chains facing similar issues.

The case in point considers the view of ease of operational deployment, performance in terms of energy savings and overall cost effectiveness in the production of a unit that may not only address immediate housing supply needs, but also to afford a quality of construction that may ensure a longer life cycle of either a single unit or a cluster of units in a housing agglomeration^56–58^. A variation of a multicriteria integrated analysis approach has been used to take into consideration the parameters mentioned, followed by a post occupancy evaluation of the results. The demonstration project also looks at customization of design and spatial flexibility based on a kit-of-parts that allows for a variety of design options in terms of both a single unit or multiple units^59^.

### Analysis and taxonomy of prefabricated structures

The scale and ways in which component fabrication and assembly of prefabricated housing systems may be taxonomized, as Boafo et al.^34^ suggested, may be by categorizing the structures of the four main prefabricated building concepts in terms of their unique parts, namely, Components, Panelised structures, Modular structures, and Hybrid structures. Specifically, in the case of the Modular type, the structure is produced and assembled off-site, either as a single unit or as a small number of different components that can be assembled in a prescribed way^34^. Concerning the Prefabricated Elements type, the unit may be put together either on site or at the fabrication plant by compiling a kit-of-parts that may then be assembled according to the preferred locale or as circumstances allow. In the case of the Container generated type, the unit may also be built on site or off-site, with possible layouts relying on reconfigurations of standard shipping containers.

Following this classification, Savvides et al.^27^ proposed a framework for mapping the diverse collection of prefabricated housing units, by proposing a taxonomy based on available literature and on a review of published or otherwise available examples and case studies. These samples are important in the assessment and categorization of prefabricated housing unit systems in terms of characteristics of common architectural components. The idea behind this attempt of coming up with a taxonomy in terms of archetypal families of units that entail similar components and assembly and organizational characteristics, was not to juxtapose these characteristics, but rather to critically present the effectiveness of the various alternatives as they may also satisfy and serve the “green” dimension in a broader design framework for the prefabrication of residential structures.

Even though the literature on the subject is limited, there are a number of studies relating prefabrication in housing with energy efficiency. Savvides et al.^27^ examined several case studies (14 modular, 30 prefabricated elements, 6 containers generated), and identified 46 types that exhibit distinct prefabrication characteristics as these are indicated in part in Table 1^27^.

**Table 1:**
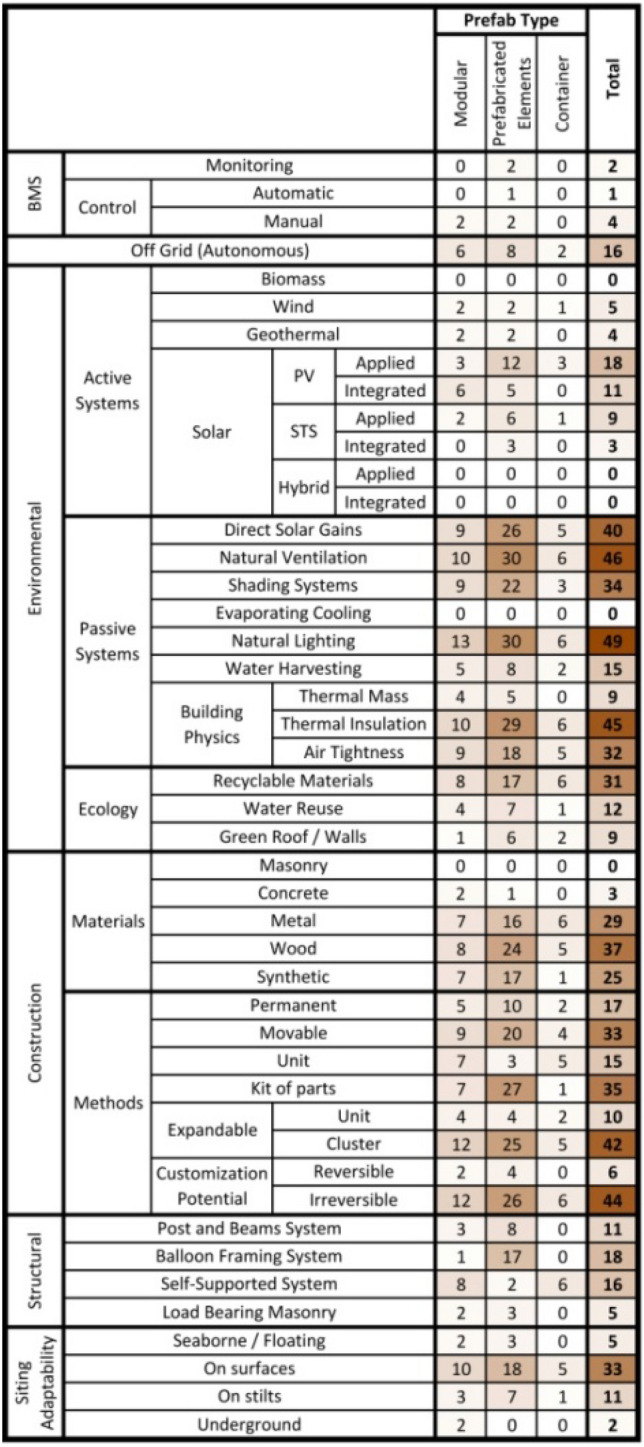
Comparative table between the characteristics of all types of prevalent prefabricated housing typologies^27^.

It is evident that the most popular type of units (that may also be used off-grid if required) is the prefabricated elements type, followed by the modular type and the container type. This could be explained because the use of prefabricated elements can be more affordable due to an easier production process, when compared to the other two types, which have more limitations in that field, as they are not very easy to produce and build. Therefore, regarding construction, one of the prerequisites for assembly is that in whole or in part a typical unit should lend itself to being transported. Transportation could be facilitated, if it were to be broken down into separate components, wholly or partially assembled at the factory, and transported to the construction site for assembly completion and erection. Furthermore, if it were to be modular in conception, it would offer the possibility of being organized into clusters of many units, which themselves could help make up a larger, more extensive complex. Furthermore, it could offer the possibility of being able to operate off-grid, given the integration of such components as solar systems, chemical sanitation fixtures, water gathering infrastructures, etc.

The construction materials palette could include a variety of single material or composite components made from metal, timber, masonry and/ or hybrid materials. The associated structural system configuration would most likely be based on post and beam assemblies or a balloon framing configuration or even a self-supported load bearing system. In terms of the siting adaptability of the unit, this should be able to be placed either on pads or on stilts.

In most of the case studies, significant consideration is given to the inclusion of passive systems that look to secure and provide natural lighting and ventilation, while shading systems regulate the unit’s performance in terms of solar gains and heat losses by the incorporation of thermal insulation and by securing air tightness of the building envelope, especially where thermal bridges may occur. Furthermore, if the unit assembly helps reduce the heating and cooling energy loads, it may reduce the operational costs and contribute to the thermal comfort of the users.

Subsequent requirements were for the units to have an active renewable energy system to regulate the energy balance of the building, by means of energy production autonomy. Specifically, the research tends to indicate that this goal could be achieved through the strategic use of specifically applied, integrated photovoltaic panels (PVs) or applied solar thermal systems (STSs). Finally, even though ecological practices are not met in all the case studies, concern with good practice in this regard is becoming increasingly popular, as it contributes to the minimization of the ecological footprint of the building by using recyclable materials and green roofs and/ or walls, as well as by incorporating mechanisms for water reuse and local rainwater storage. Ultimately, these findings may constitute an initial roadmap as to the choice of components and assembly techniques and related infrastructures that can kickstart schematic design and constitute primers for design development and construction specifications.

## Methodology

The proposed investigation is based on a research by design approach. It begins by reviewing relevant references outlined in the previous section in terms of unit design, fabrication, and assembly, while aspects of environmental responsibility have also been examined, through two more research prongs.

In the first prong, the review of the literature leads to a narrowing down of relevant typological precedents whose parameters then influence morphological attributes. These attributes are matched to specific locational characteristics, which rely on climatic, cultural and economic attributes and which influence various architectural components of the design and construction documentation and play a role in the value chain of fabrication and assembly^60^.

In the second prong, the conceptualization of the demonstration project illustrated, deals with the optimization of the prefabrication process as it exists in Cyprus and in a way that adheres to proven principles of environmental design and cost effectiveness. The proposed housing unit was also evaluated in terms of energy and daylighting performance and cost savings. Daylighting simulations were performed to explore the unit’s daylight autonomy, annual sunlight exposure and glare probability, with the ultimate aim of defining the installation of shading devices, while a cost analysis was carried out aiming to validate the affordability of the unit. Passive design strategies played an important role in the examination of the design proposal for this pilot project. These were quantified and verified using an officially sanctioned software called iSBEM CY (Simplified Building Energy Model, iSBEMcy_v3.4.a), which was developed for this purpose by the Republic of Cyprus and with a view to maximizing energy savings as a result of the building’s configuration and the attributes of the selected building components, while at the same time ensuring the thermal comfort of the building’s occupants.

### Daylighting performance; simulation settings

For carrying out computational simulations for the daylight performance of the prefabricated unit, the plugin “DIVA” in Rhinoceros software was used. ‘DIVA’ uses the Radiance simulation engine which is widely accepted and validated for lighting simulations. The 3D model of the prefabricated housing unit was designed in the Rhino 7 interface. The parameters set, were that the occupation pattern would be in a time range from 08:00 to 18:00 (a range that includes daylight hours for Cyprus), using Daylight Saving Time (DSM). The analysis does not include night-time hours during which artificial lighting would be required. The simulation was carried out at a work plane height of 0.85 m above the finished floor, using a grid of 0.20 m × 0.20 m for a more detailed analysis of the daylight distribution. Additionally, the materials’ surface reflectance is provided in Table 2.

**Table 2:** Surface reflectance values of the materials used.

Layer—objects	Surface reflectance	Layer—objects	Surface reflectance
Walls	0.70	External floor—Veranda	0.40
Interior floor	0.40	External roof—PV-glass	Tvis: 0.47
Roof	0.80	External roof—PV-solar cells	0.20
Furniture	0.50	Shading devices	0.30
Window—glass	Tvis: 0.65	Ground environment	0.20

**Figure 1:**
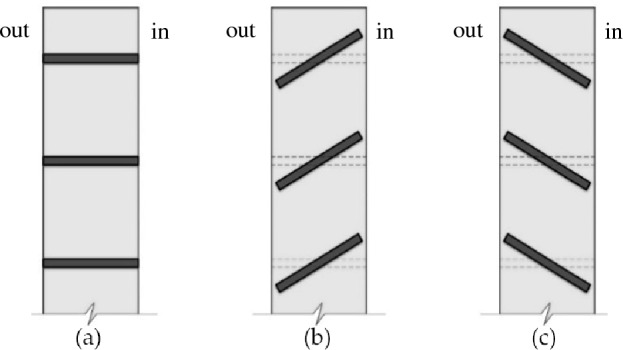
Rotation of shading devices; (**a**) 90°, (**b**) 60° (30° counter clockwise), (**c**) 120° (30° clockwise) slats rotation.

Additional considerations included the high solar radiation levels in Cyprus, which prompted the study and implementation of shading devices to the unit. Consequently, the use of horizontal shading devices is taken into account, with three different rotational positions. Different rotations were essential to compare shading configurations and decide on the optimal setting based on the orientation of the various program elements. The positioning and use of shading slats devices are shown in Fig. 1.

### Construction costs comparative analysis

Five different scenarios were examined and compared in terms of the primary construction materials and methods, to estimate the potential construction cost:

- Steel-frame Construction with Eco Panels Masonry
- Steel-frame Construction with Light-weight Masonry
- Timber-frame Construction
- Concrete Masonry Unit (CMU) Construction
- Reinforced Concrete Construction (Conventional Construction)

Some assumptions were made for the juxtaposition of the different scenarios to be valid and realistic in terms of both a robust bill of quantities and informed costs.

First, the size of the unit is considered to be constant for all cases in order for the bill of quantities to eliminate errors in comparisons. Second, for all the alternative cases a structural system analysis was carried out in order for the sizes of the respective cross sections of the load-bearing components to be established.

For steel-frame construction with light-weight masonry, the sizes of the beams and columns used, as well as the thickness of the slabs, were the same as the ones used in the demonstration project. For the other alternatives, the analysis revealed the following:

With regards to timber-frame construction, the wood members selected for the roof measured 6.5 × 10 cm, while the total thickness of the masonry frame was 18 cm. The analysis also recommended that two metal columns with a square section of 10 × 10 cm should be used.

Following the same methodology, the analysis for CMU Construction indicated that a metal column with a section of 10 × 10 cm should be used, so as to reinforce block construction. Similarly, an additional measure might be the use of reinforced concrete applications, 15 cm in height and sited atop all masonry openings (windows and doors).

Finally, as far as conventional construction is concerned, all beam sections measured 25 × 50 cm, with six column sections at 25 × 25 cm with foundation and roof slabs of 35 cm and 17 cm thickness, respectively.

## Design process

Having thus defined the design parameters and specifications, the demonstration project aims to provide an improved and more holistically considered alternative for a prefabricated housing unit, based on a standardized kit assembly and on environmental design principles that are governed by an integrated design approach. A detailed description of the proposed research by design is given below, focusing on the key design decisions that govern the demonstration project.

### Design objectives

Overall, the model is governed by the following characteristics, which are based on the outcomes of the taxonomy presented in “Analysis and taxonomy of prefabricated structures”:

- With regards to aspects of construction, the unit should lend itself to portability in whole or in part based on the assembly of larger building components from a standardized kit of parts, offering flexibility in configuration but also plurality in terms of spatial layout and design, with the possibility for future expandability also taken into consideration. The construction materials should be chosen amongst metal, wood and hybrid materials taking into consideration their thermal properties and reuse potential.
- Concerning the structural system, the research indicates that a post and beam system lends itself well as the main structural system for this demonstration project, in a balloon framing configuration or even as a load bearing, self-supported system.
- In terms of conforming to and managing physical site adaptation, the proposal is for structural pads or stilts to negotiate siting challenges resulting from uneven ground.
- With regards to the environmental design dimension, the research indicates that the unit’s active energy systems should include applied or integrated photovoltaics (PVs) and/ or applied solar thermal systems (STSs)^61^, at least for Cyprus and its Mediterranean climate and insolation prevalence.
- The unit’s passive systems with regards to securing natural lighting and ventilation should be utilized to the highest possible degree. Other considerations allow for shading and the regulation of direct solar gains, when and where required, and the provision of thermal comfort for potential users through the use of appropriate layers of insulation and minimization of heat loss by designing for an airtight unit.
- At the same time, reduction of energy requirements that result in energy savings should be pursued by integrating smart building management systems.
- Lastly, utilizing recycled or reused materials in the construction will render the unit friendlier to the environment.

### Architectural design

**Figure 2:**
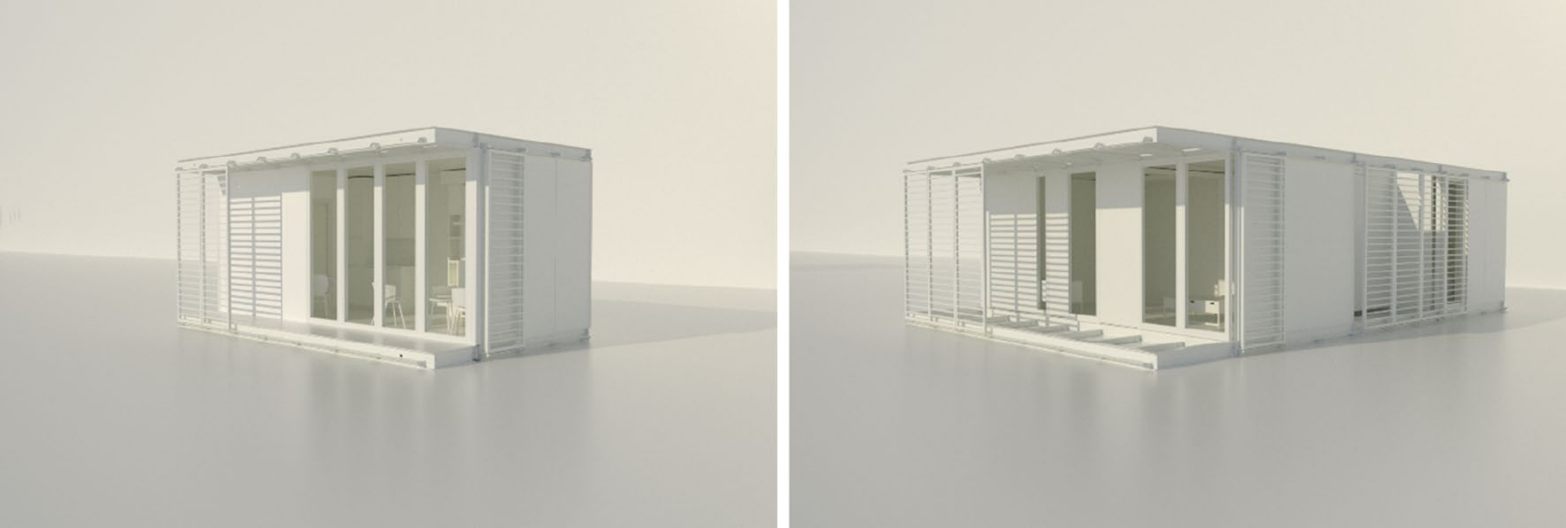
Core unit (left), demo building (right) comprising by the unification of two core units connected to each other by an internal courtyard, resulting to a 60 m^2^ house.

With the key design strategies and design parameters outlined above (“Design objectives”), the process of investigation continued with an exploration of the possible architectural morphologies that could be adopted, aiming at a low-cost construction cycle, with simplicity of assembly and also considering efficiencies in energy requirements with low operational costs, based on a core unit of 21 m^2^ (internal dimensions of 7.00 × 3.00 × 2.70 m) consisting of a seven by three grid (7 × 3) at 1-m intervals (Fig. 2).

**Figure 3:**
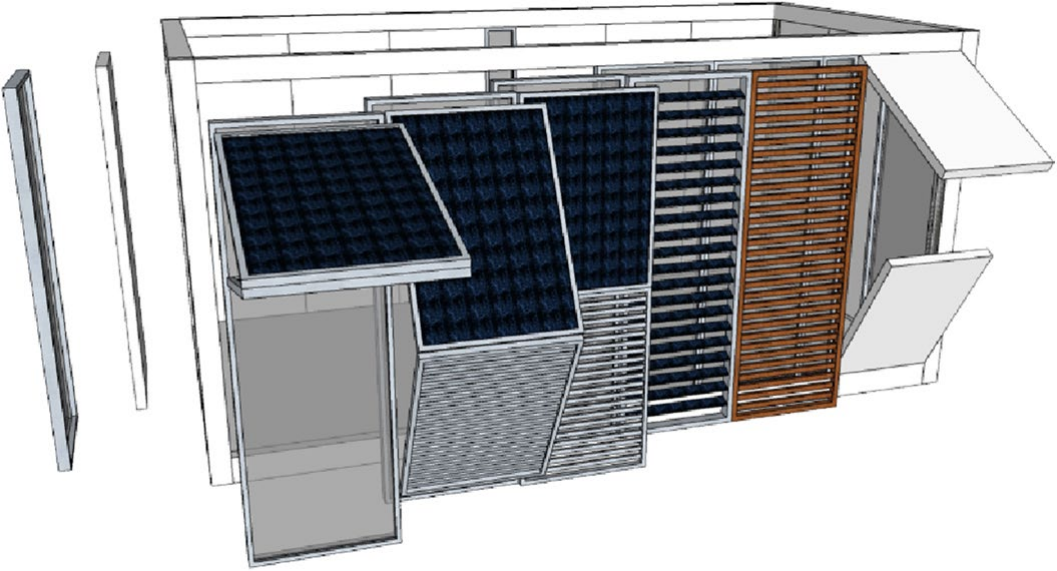
Illustration of the shell of the unit; formed by assembling panels.

The mapping of the industrial know-how in Cyprus in this industry sector (“Prefabrication use: the case of Cyprus”), it was indicated that the unit should be movable and contingent upon a kit of parts. Taking into consideration that the shell of the unit is formed by mounting panels (80 mm-thick Eco Panels), the team investigated the possibility for these to be installed and uninstalled in a straight-forward manner (Fig. 3).

This shell design option offers optimal energy efficiency performance and keeps the overall construction costs within the prescribed parameters. The installation of appropriate overhangs is recommended, as these will also act as receptors for the solar (photovoltaic and/ or solar thermal) panels. In the proposed unit’s perimeter, the study team installed drilled solar protection panels with their slat’s angle defined by daylighting performance and the results of which are presented below (“Passive systems”).

### Construction methods

**Figure 4:**
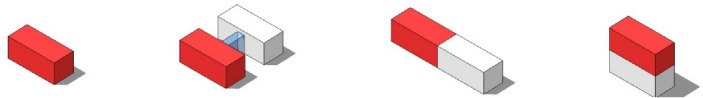
Alternative assemblies for the demonstration project. The development of the prototype is based on the second configuration from the left.

The structural design study confirmed the possibility of the proposed unit to expand in both the horizontal and vertical axes up to two floors, augmenting the possibility of adapting to alternative layouts, which could result in a unit of either 40 m^2^ or 60 m^2^ and so better serve the spatial and organizational requirements of future inhabitants, while also allowing the formation of unit clusters. For this to be achieved following the post and beam type construction, a structural analysis was performed and an outline for a “kit-of-parts” was developed that consists of seven main parts. These may be configured in several different layouts resulting in a single unit, a two-floor unit or even a cluster of units, according to the needs and requirements of the end users (Fig. 4).

**Figure 5:**
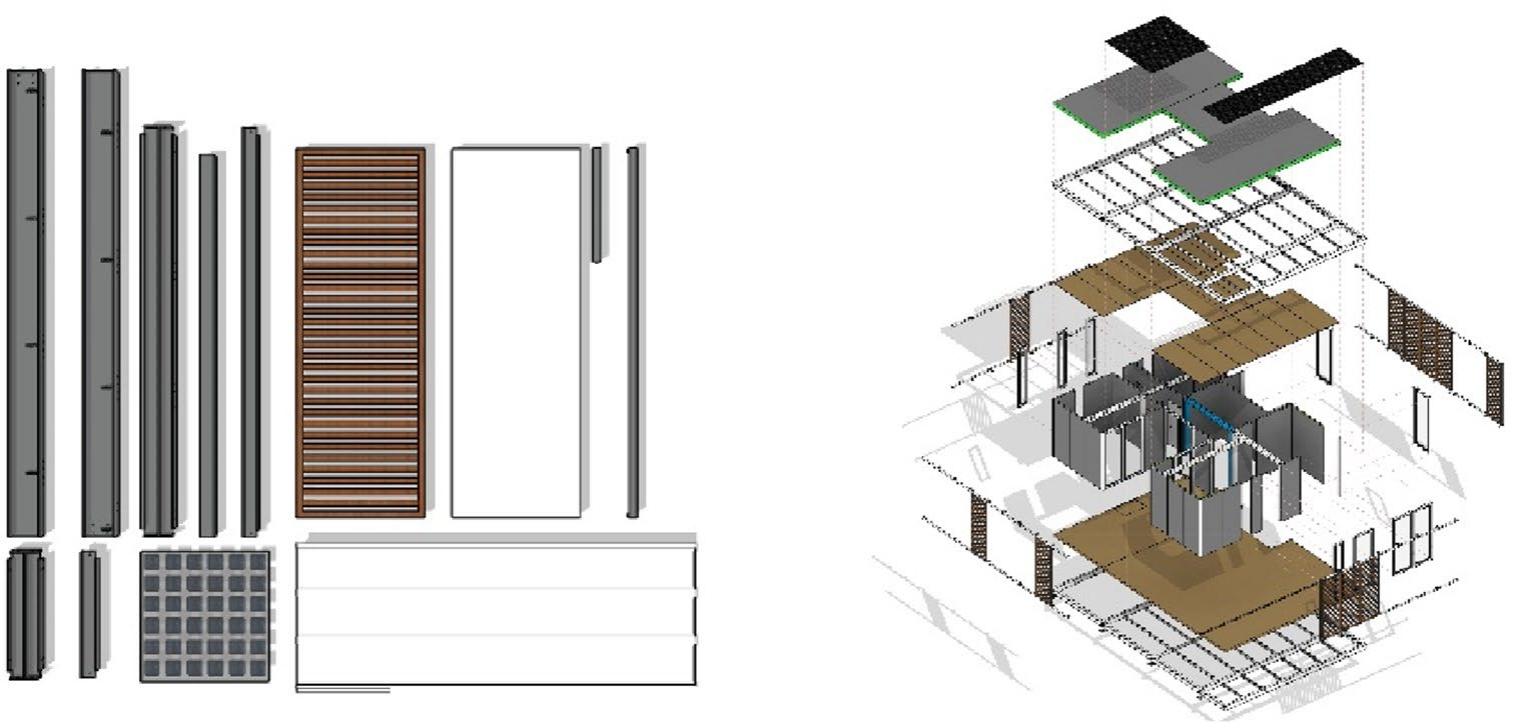
Breakdown of "kit-of-parts".

The sets of components specially configurated for the design proposal form three element groups: (a) the primary structural elements, based on steel profiles IPE220 and SHS 100 × 100 × 8, (b) the secondary structural elements, based on steel profiles IPE100, and (c), any secondary structural elements, utilizing lightweight steel to act as a support for the wall structures and the framing of any openings (Fig. 5).

The structural elements are fabricated and assembled into the core unit in a controlled environment off-site. The core units are then transferred to the site for further assembly. The demonstration building was fabricated and sited on a flat lowland field in the outskirts of Nicosia, where it will remains for a time as an exhibit, serving research and commercial, promotional and industrial demonstration purposes.

### Environmental design

Along with all the previously mentioned strategies, the design team performed an evaluation on the various energy and environmental design initiatives, which could be incorporated in the demonstration unit in conformance to bioclimatic design principles. This evaluation covered heating and cooling strategies for improved microclimatic conditions; daylighting strategies aimed at creating ideal visual comfort conditions indoors; and meeting the goal of minimizing energy of consumption and significantly reducing operating costs.

#### Active systems

In the design development stage of the process, parameters that had to do with reaching a zero or nearly zero energy requirements building were incorporated, and it was deemed useful to consider the architectural integration of photovoltaic and/ or solar thermal systems early on. The above would cover the energy needs of the unit in case it is required to operate autonomously off-grid. This mode of thinking led to the selection of a building-integrated and opaque PV system on the unit’s roof, while atop the south facing overhangs, a semi-transparent PV building-integrated system was chosen, which also passively serves as a shading device for the deck below.

#### Passive systems

**Figure 6:**
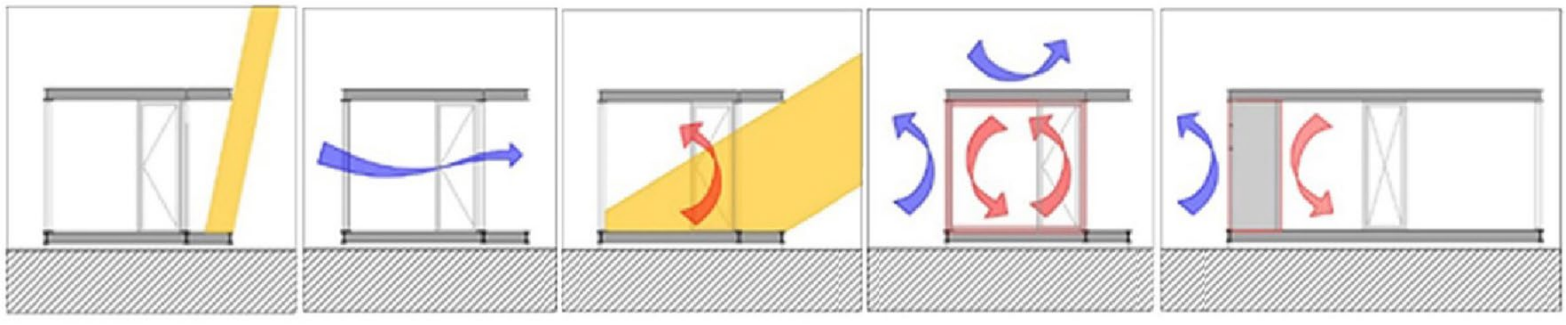
The main passive strategies applied to the unit. From the left to the right: passive shading system, natural cross ventilation, direct solar gains, thermal insulation, buffer zone.

Several strategies also emerged in the context of passive design (Fig. 6), where savings in energy requirements and securing the thermal comfort of the inhabitants was another main goal. Natural lighting is achieved with the strategic placement of the openings, to make use of the best insolation and shading conditions, while cross ventilation is achieved by the strategic location of openings on the building shell. This approach works well with the anticipated cooling strategies that operate in conjunction with the envisioned passive shading systems.

In considering heating strategies, the unit’s design allows for direct solar gains and thus reduces the mechanical heating loads in the cool and cold periods of the year, while in the warm and hot periods, the integrated passive shading systems help prevent—or at least minimize—over-heating of the interior habitable spaces. At the same time, the aim of achieving overall shell air tightness becomes more feasible through the placement of a thermal buffer zone on the north side of the unit with the parallel use of appropriate layers of thermal insulation which minimize unwanted thermal exchanges with the outside environment.

#### An environmental and sustainable design approach

As mentioned earlier, by incorporating sustainable design practices in the overall unit fabrication and configuration, the study team tried to maximize the use of recycled structural parts and construction materials in the assembly line, especially those from aluminium or wood. Recycled elements, mostly from Oriented Strand Board (OSB), were also used for fixed and movable furniture including interior finishes, such as, flooring and ceiling treatments and also exterior cladding finishes. This is in line with the broader philosophy governing the expected simplicity in the assembly and disassembly of the proposed unit, which revolves around ease of transportation and the reconfiguration of kit elements. These in turn, are derived from modular and interchangeable components, so as to address the ever-changing needs of potential users. This strategy also supports the unit’s ecological design approach, achieved by accompanying the use of the aforementioned kit-of-parts with related dry construction techniques.

### Automation systems and software

Aspects of “smartness” are addressed by the installation of sensors which constantly record relative environmental parameters, passive elements (openings and overhangs) as well as heating, cooling and lighting components. The installation of an automation system is for purposes of regulating and taking over the unit’s energy management so as to optimize operational efficiencies and to reduce losses to a minimum. Another element that defines the demonstration project is the incorporation of a passive interaction user interface, which updates a built-in user profile based on recording behavioural patterns.

## Discussion of the results and conclusions

As presented in the methodology chapter, the proposed demonstration project was evaluated in terms of energy performance, daylighting performance and potential cost savings related to making informed decisions in utilization of materials and adopted methods of fabrication and assembly and on streamlining building operations.

### Energy performance

The local rating system looking into energy performance assigned a grade “A” to the unit, which is the highest possible mark, and which constitutes a legally binding designator in Cyprus. That makes the unit a nearly Zero Energy Building (nZEB), with low operational overheads and maximum energy efficiency.

**Figure 7:**
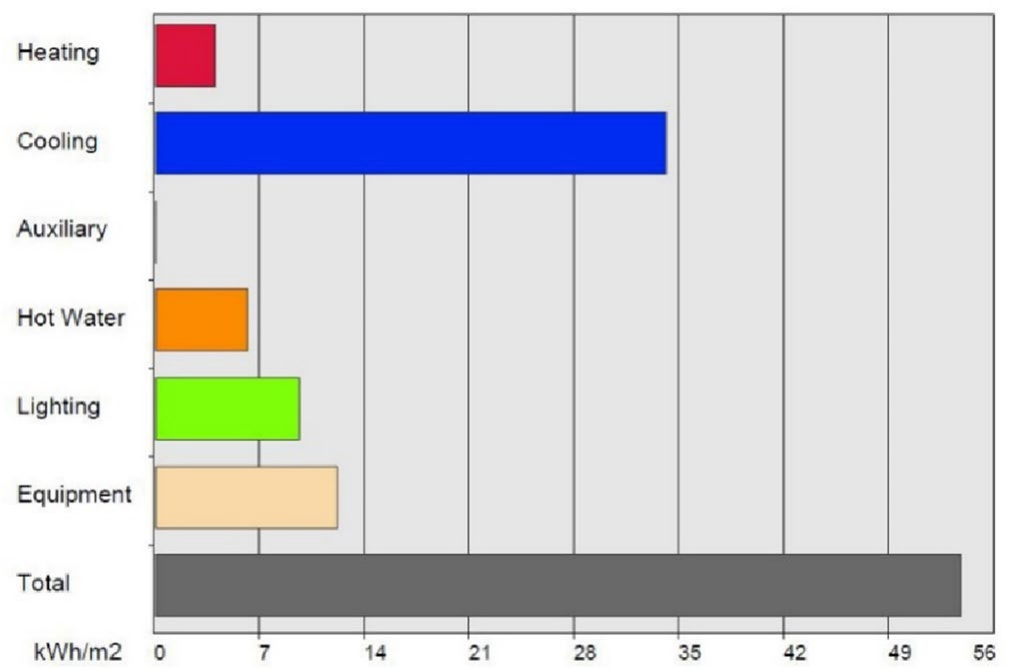
Analysis of the energy consumption of the proposed building, exported from the software.

According to the simulations, its total energy consumption is 53.7 kWh/m^2^/year, which is covered in its entirety by the Renewable Energy Systems (RES), 95% by the Building Integrated Photovoltaics (BIPV) and 5% by the Solar Thermal System (STS). An analysis of the loads with regards to energy consumption is presented in Fig. 7.

**Figure 8:**
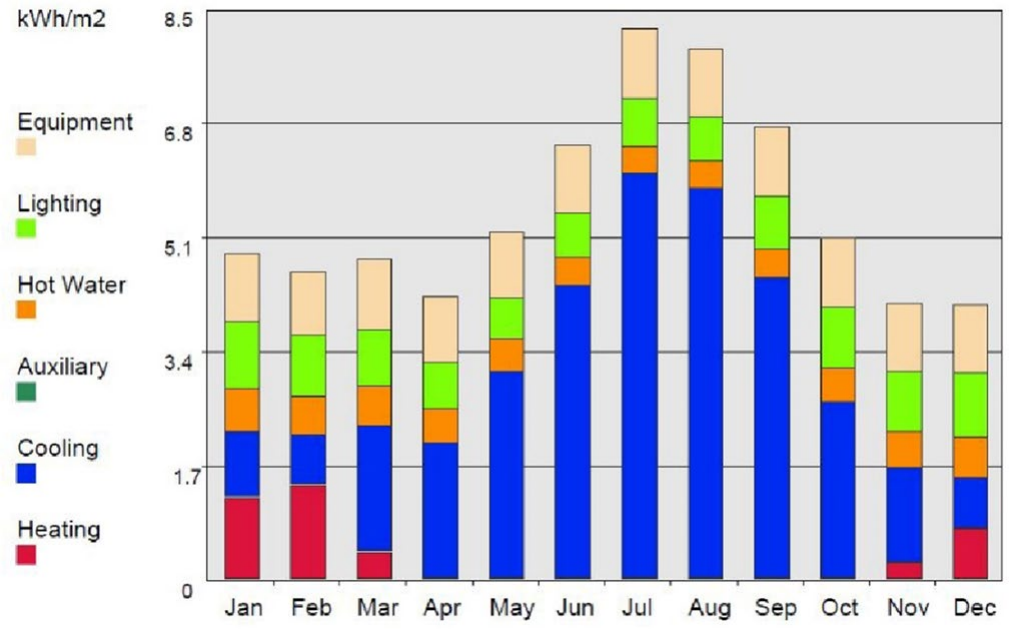
Energy consumption loads of the proposed building, analyzed per month, exported from the software.

As shown in Figs. 7 and 8, the cooling loads of the unit are significantly higher than the heating loads throughout the year, a fact explained when considering the unit’s floor area as a ratio of the significant extent of the south-facing glazed façades of the unit. The resulting analysis supports the strategies used by the research team with regards to environmental design decisions. It also supports decisions concerning choices in the techniques and materials used for construction and in meeting the mandate of ensuring natural cross ventilation resulting from passive cooling strategies.

### Daylighting performance

Daylighting analysis was also considered an important factor in meeting the environmental design parameters for the prefabricated unit. Even though the optimal lighting levels for a residence cannot be defined with absolute values—since the tasks and activities differ for each user based on their needs, simulation methods to study daylight performance were used. Based on those, shading devices were identified to maintain acceptable natural lighting levels and at the same time reduce unwanted solar heat gains. Daylight performance using static metrics was limited to specific times and days of the year and so it was that in the current research, the dynamic metrics of Daylight Autonomy (DA), Continuous Daylight Autonomy (cDA), Annual Sunlight Exposure (ASE) and Useful Daylight Illuminance (UDI) were used. The minimum illuminance level was set at 300 lx^62,63^.

**Figure 9:**
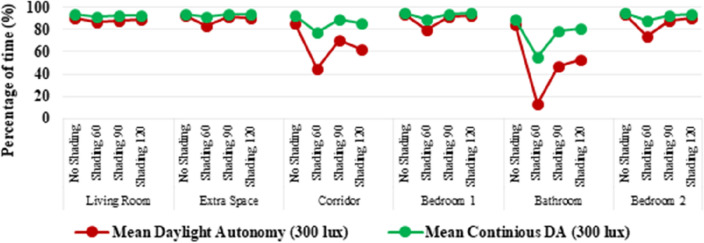
Mean daylight autonomy and mean continuous daylight autonomy.

Considering that the Daylight Autonomy is measured as the percentage of the occupied area that can achieve 300 lx or more, for more than 50% of the time that the space is occupied^64^, the findings shown in Fig. 9 indicate that all proposed rooms have sufficient access to daylight during the times the unit is occupied. Calculations show that the mean continuous daylight autonomy has higher results than the mean DA.

**Figure 10:**
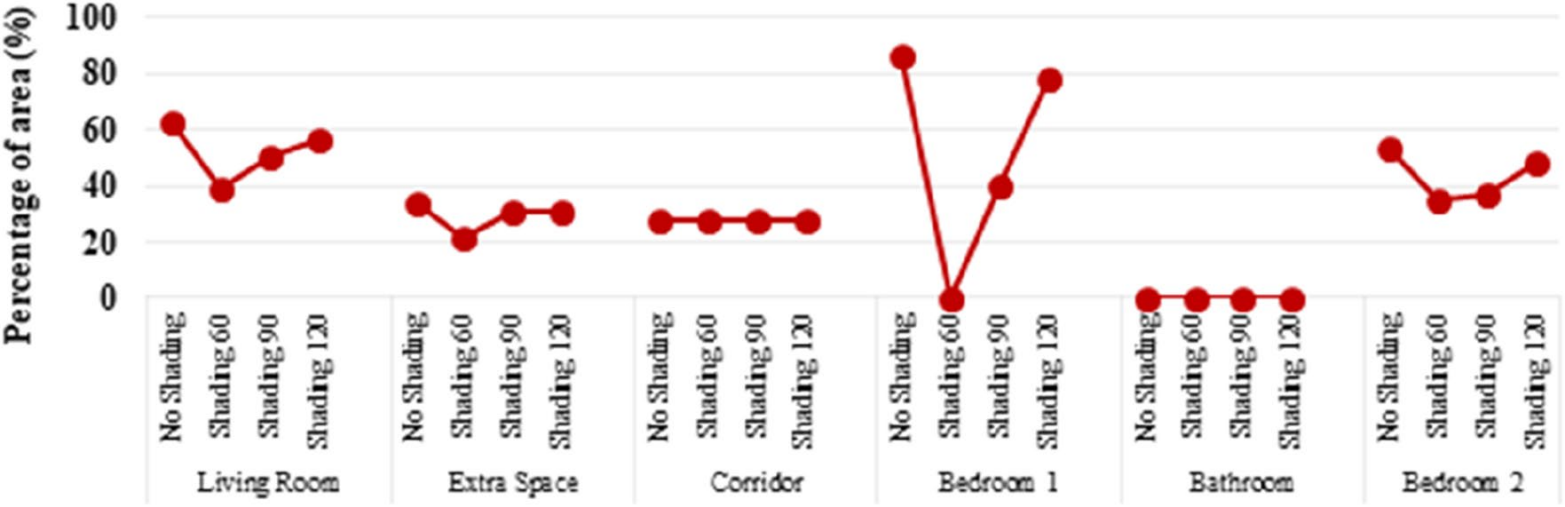
Annual sunlight exposure.

In addition to Daylight Autonomy, the Annual Sunlight Exposure metric is used to study the exposure of a space to direct sunlight. This indicator is usually expressed as the percentage of a space that achieves 1000 lx of sunlight (the ambient bounces in radiance parameters is set to 0) for more than 250 h per year. LEED and the WELL Building Standard^65^ require that the ASE does not exceed 10% of the room’s area^64^, The WELL Building Standard V1, 2014^66^. Figure 10 shows the findings for ASE for the prefabricated housing unit. It seems that all spaces have significant exposure to direct sunlight due to large glazing areas. The use of shading devices reduces the area exposed. As shown in Fig. 8, the best option for all the rooms in the prefab unit is using louvre slats at an angle of 60°, which produced ASE results of less than 40%. Additional internal shading options, such as blinds or curtains, could further protect from sunlight exposure.

**Figure 11:**
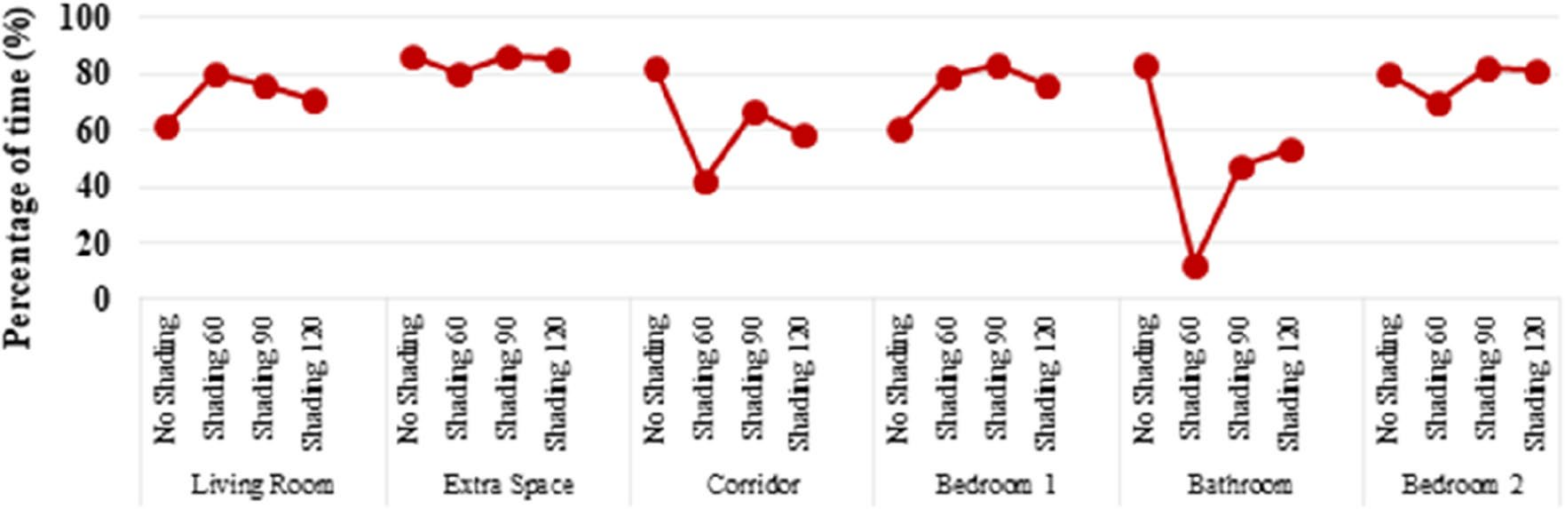
Useful daylight illuminance (300–3000 lx).

Another dynamic indicator widely in use is the Useful Daylight Illuminance. The data presented in Fig. 11 indicate that all four spaces in the unit have more than 60% of their area with useful daylight levels (UDI 300–3000 lx). When using shading devices, the UDI between 300 and 3000 lx is increased up to 80%. This metric is another verification that mounting shading devices, especially in the Living Room and in Bedroom 1, could greatly improve the visual comfort of the users.

**Figure 12:**
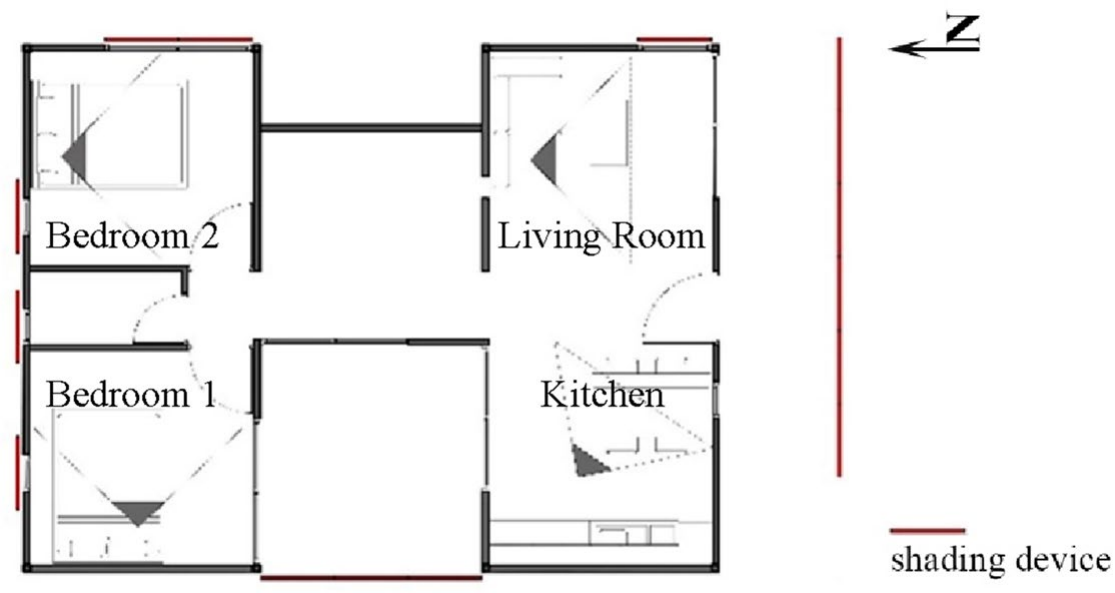
Viewing positions for glare probability analysis.

Another important aspect of daylight performance considered for investigation is glare probability. For this analysis, the static metric of DGP was used. Since there are several viewing possibilities for each room, four options were chosen, as shown in Fig. 12, to analyze glare based on relative occupant position during different activities.

**Figure 13:**
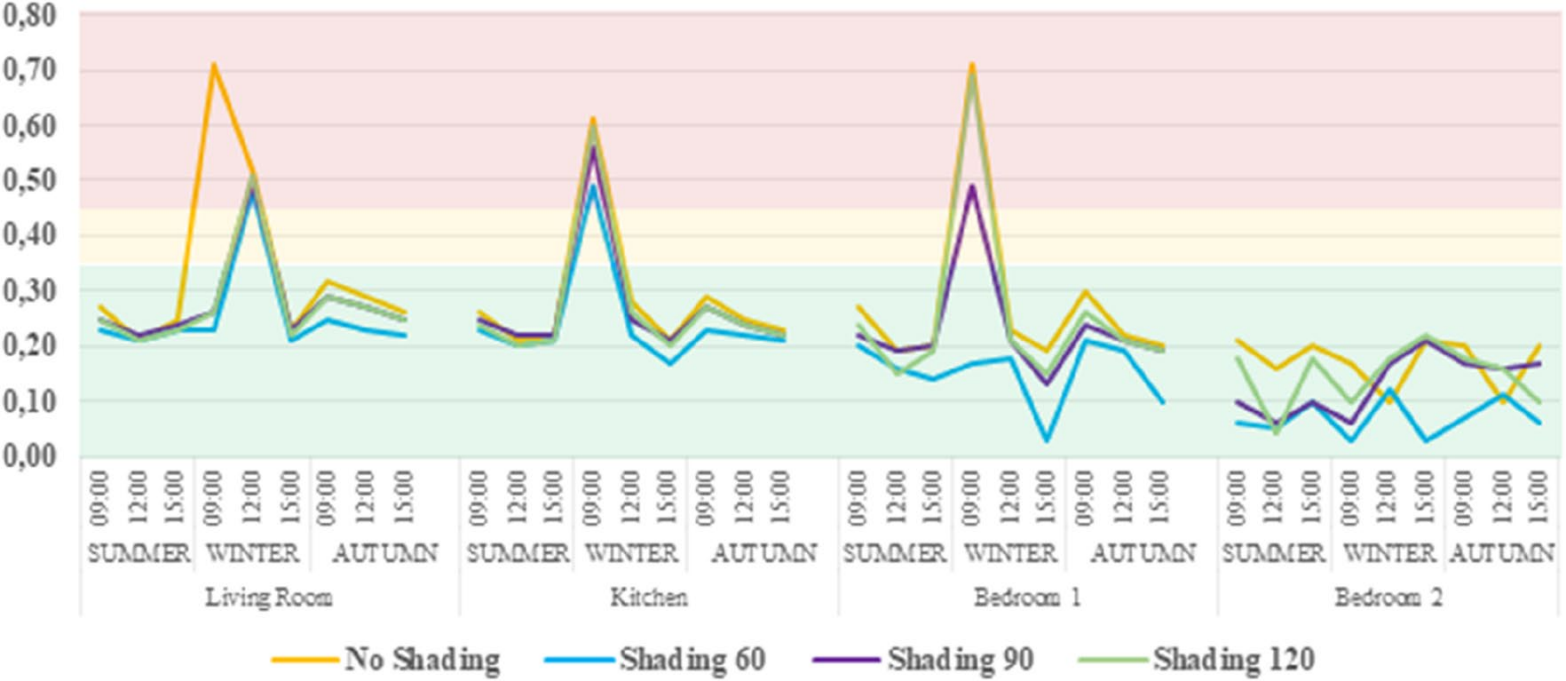
Daylight glare probability (DGP) analysis.

The analysis was carried out for the summer solstice, the winter solstice and the autumn equinox at 9:00, 12:00 and 15:00. The data show that when no shading devices are used in the Kitchen, the Living Room and in Bedroom 1, glare issues could appear especially during winter and specifically around 09:00, since at that time the sun is at a lower altitude. When shading is implemented, the glare probability is reduced below 0.45 or even 0.35, which is defined as imperceptible. The slats’ rotation of 60° provides the best protection from glare for all rooms (Fig. 13).

In conclusion, the static and dynamic indicators presented above indicate adequate to good daylight levels throughout the year, while installing shading devices (especially when set at an angle of 60°), can reduce the glare effects substantially.

### Cost analysis

The purpose of the cost analysis was to explore different construction methods, mostly in terms of alternative materials and options concerning the construction of the demonstration project. Specifically, the unit under investigation is set as a studio type covering an area of 30 m^2^ (including the covered veranda). The typical formation of this unit consists of a steel frame construction and Eco Panels for masonry and roofing. The juxtaposition of the different scenarios revolved around the relevant construction costs and the mechanical properties of the primary construction materials.

#### Scenario-based construction costs

After the design parameters were established, all quantities for the building components and surface treatments were calculated in order for a bill of quantities to be created. A bill of quantity was used for each case study/ building scenario. The bills included an expanded list with regards to the foundation, the load-bearing components of the building’s structure, the roof and flooring, the masonry, insulation, paints and coatings (e.g. plaster skim-coating when necessary). All sub-contractor works including plumbing, electrical services, tiling and equipping were not included on the bill of quantities, and therefore the final cost estimation for each study is not the real cost of construction. These unaccounted-for costs were considered to be on the same basis, similar for all case studies, and in accordance with the prices in the Cypriot Construction Industry Market at the time of this study. When all five bills of quantities were completed, the costs for each construction method were estimated, as presented in Table 3.

**Table 3:** Estimated cost per 30 m^2^ unit.

Type of construction	PreFab unit	Light-weight	Timber-frame	CMU	Conventional
Cost (€)	8773. 51	13,125.20	10,170.64	13,292.56	13,223.53

The exploration of alternative construction methods revealed that the demonstration project presents the most inexpensive option to build as opposed to other steel-frame, timber-frame and conventional constructions, a fact which supports decisions made on merit for the proposed unit.

The cost gap lies in small differences presented in the construction methods reflecting the structures themselves. The following table (Table 4) describes the relevant costs analytically. What is apparent is the efficiency in the prefabrication approach selected for the demonstration project, as no coating (and in particular no plaster-skim coating) is necessary, which is a consistent factor that raises the costs in the alternatives. Another factor raising costs is the necessity for a slab foundation, which was not required in the demonstration project.

**Table 4:** Construction cost analysis; prefab unit vs conventional construction vs light-weight construction vs timber-frame construction vs CMU construction.

Type of construction	PreFab unit	Conventional	Light-weight	Timber-frame	CMU
Costs (€)					
Foundation	–	1695.00	2839.80	2839.80	2839.80
Structure	4610.81	4916.81	4610.81	653.00	2531.60
Roof	1921.50	1475.80	1472.46	2284.18	1421.80
Floor	1428.00	801.74	801.74	801.74	801.74
Masonry	813.20	1845.30	3591.20	3591.20	1532.30
Coating & paints	–	2698.08			2994.28
Total (€)	8773.51	13,223.53	13125.20	10,170.64	13,292.56
Total (€/m^2^)	292.45	440.78	437.51	339.02	443.09

What is also of note is the cost difference per square meter when comparing different scenarios. The demonstration project is €148.33/m^2^ cheaper than conventional construction, whereas the difference between the unit in question and the timber-frame alternative drops to €46.57/m^2^ (Table 4).

Findings indicate that the proposed construction method and materiality of the demonstration project shows definite advantages over the alternative types of construction as it is more efficient and affordable. Not only are there benefits during the construction works, but renovation costs seem to be low as well, especially when the life span of the materials is taken into account. The only drawback of the proposed unit could be the amount of CO_2_ emissions in the production stage of steel, as well as the amount of the embodied CO_2_ in the some of the component materials, especially where the eco-panels are concerned (Table 5). On the other hand, during the construction phase itself, the CO_2_ emissions, when assembling the unit, are kept to a minimum. This is due to an efficient and streamlined production line process benefitting from economies of scale.

**Table 5:** CO_2_ emissions and embodied CO_2_ in steel, concrete and timber.

PreFab unit			
Steel	Polyurethane	Timber	Concrete
CO_2_ emissions (TN)/TNIN1.9 production		0.3	1
Embodied CO_2_ 1.27 (kg CO_2_/kg)	4.26–4.84	0.4525	0.247

### Concluding thoughts

The research aim was to make a contribution to the literature by considering an actual demonstration project, which has been used as the test bed for investigation of the front end of the design and construction process where the typology of housing units is based on prefabrication and modularity. An important aspect of the work has been the collection of disparate literature references and case studies and their compilation into a taxonomy of archetypes and into groups with specific characteristics—into “families of likeminded projects”. By compiling this taxonomy, the researchers determined that the most popular type of prefabricated housing units is the “elements” type.

Using the acquired knowledge, adapting appropriate technologies, and making best use of local technical knowhow, informed decisions were made in terms of design, fabrication, transportation, assembly strategies and energy efficiency from the early stages of component production to on-site assembly. Furthermore, the demonstration project is intended to be able to function off-grid and to achieve maximum utilization of the efficiencies and cost savings associated with a value chain heavily reliant on using prefabrication to come up with a portable kit-of-parts of the main architectural components.

A further aspect of its originality lies in the selection of the materials palette used in designing the prefabrication process and the subsequent transportation requirements to a specific site, while managing the dimensional restrictions imposed by standard shipping, all the way down to road widths and bridge clearances. Other challenges include the staging area, the assembly sequence (on site) with the least possible components in the shortest amount of time and without utilizing specialised skilled labor.

In cost analysis, it was made clear that the kit-of-parts construction method and materiality of the demonstration project were more efficient and affordable as the research shows that the conventional type of construction will cost more than double that of the proposed method. The only drawback identified was the amount of CO_2_ emissions in the production stage of steel and the embody energy of materials. Considering energy performance, the demonstration project scored particularly well, scoring a grade A by following the iSBEM CY methodology, justifying the environmental design strategies applied, although more data should be collected to acquire an even more comprehensive picture. In evaluating movable shading systems and overhangs as design options in daylighting simulations, these were shown to be positive in terms of ensuring visual comfort, as the UDI between 300 and 3000 lx was increased up to 80% and glare probability was defined as imperceptible.

Moreover, this proposal provides an alternative way of looking at immediate and short-to-middle-term housing needs for a country like Cyprus, being at the forefront of receiving significant pressure to provide decent housing environments for the waves of international immigrants arriving to the island—and also for its own citizens in these times of economic crisis leading to hardships for households in securing access to decent housing.

Consequently, the prefabrication approach needs to be examined holistically and issues of life project feasibility and financing, as well as building life cycle performance, need to be carefully reviewed. The current levels of industrial know-how in the housing prefabrication industry on the island was a major limitation on the project. The design and construction processes have to be adaptable to industry-imposed limitations and to material supplies availability in order to achieve optimum results under the circumstances. It was due to these limitations and in pursuing the best possible outcome, that delays to the time schedule occurred.

Finally, this proposal hopes to jumpstart a review of both building codes and planning and policy frameworks that prescribe both design standards and performance criteria for alternative to the conventional housing solutions as a steppingstone towards more sustainable communities and their potentially new residential neighbourhoods or infill possibilities, vacant or underutilized lots, in existing residential quarters.

### Future research

The proposed system is defined in such a way to be easily adapted and integrated in an existing operational cycle based on production processes associated with this product the outcome of which will be savings in construction costs and timeframes of building assembly as a by product of standardization of both components and processes. Another benefit, this time to the designer, but also potentially the end user, is the expected ease and flexibility of configuration during the unit’s life cycle and its potential for expandability to create either a bigger unit or result in a cluster of units.

Post occupancy evaluation will provide the opportunity to re-examine the whole range of construction detailing documentation both at the fabrication plant and on the staging and assembly site to address all such challenges that were revealed throughout this time. In addition to that the team has already tapped onto the smart management systems monitoring the operational parameters of the demonstration project, with an eye to fine tuning adjustments that may further minimize operational costs and maximize user comfort. Specialized sensors and data loggers have also been incorporated in strategic locations to monitor the structure’s environmental behavior and energy producing potential. The final design may then be evaluated with a greater degree of certainty and a more accurate cost estimation of the unit will be possible. The next stage of investigation also includes the spatial possibilities provided by the unit when organized in clusters thereby exploring the unit’s versatility and adaptability as contrasted to more conventional and static housing developments.

## Data Availability

All data will be available on reasonable request from corresponding author.
